# Investigation of Anti-*Toxocara* Antibodies in Epileptic Patients and Comparison of Two Methods: ELISA and Western Blotting

**DOI:** 10.1155/2013/156815

**Published:** 2013-04-22

**Authors:** Mohammad Zibaei, Farzaneh Firoozeh, Parviz Bahrami, Seyed Mahmoud Sadjjadi

**Affiliations:** ^1^Department of Parasitology and Mycology, School of Medicine, Lorestan University of Medical Sciences, P.O. Box 381351698, Khorramabad, Iran; ^2^Department of Microbiology and Immunology, School of Medicine, Kashan University of Medical Sciences, P.O. Box 8715988141, Kashan, Iran; ^3^Department of Neurology, School of Medicine, Lorestan University of Medical Sciences, P.O. Box 381351698, Khorramabad, Iran; ^4^Department of Parasitology and Mycology, School of Medicine, Shiraz University of Medical Sciences, P.O. Box 713451735, Shiraz, Iran

## Abstract

The relationship between *Toxocara* infection and epilepsy was previously demonstrated by several case-control studies and case reports. These previous studies were often based on the enzyme-linked immunosorbent assay (ELISA) using *Toxocara* excretory-secretory antigens, which are not specific due to cross-reactivity with other parasitic infections such as ascariasis, trichuriasis, and anisakiasis. An immunoblot analysis is highly specific and can detect low levels of *Toxocara* antibodies. Therefore, this assay may be useful in the identification of toxocariasis in epileptic patients. We examined patients who had epilepsy and healthy subjects for seropositivity for *Toxocara* infection by ELISA and Western blotting. Out of 85 epileptic patients, 10 (11.8%) and 3 (3.5%) persons exhibited *Toxocara* immunoglobulin G (IgG) antibodies responses by ELISA and by both techniques, respectively. Moreover, in the healthy group (*n* = 85), 3 (3.5%) persons were positive by ELISA, but none was detected by Western blotting. This study indicates that *Toxocara* infection is a risk factor for epilepsy in Iran. These findings strongly suggest the need to perform Western blotting immunodiagnosis, as well as the ELISA using *Toxocara* excretory-secretory antigens, to improve diagnosis of human toxocariasis in patients with epilepsy.

## 1. Introduction

Human toxocariasis is a zoonotic parasitic disease caused by migration of the nematode worms *Toxocara canis* (dog) or *Toxocara cati *(cat) larvae to the organs and tissues of animals and birds. Human infection occurs when *Toxocara* eggs containing infective larvae are accidentally ingested or through consumption of raw or undercooked meat and giblets [1]. The larvae hatch in small intestine and migration through somatic organs, preferably the liver, brain, and eyes, and cause at least three syndromes: visceral larva migrans (VLM), ocular larva migrans (OLM), and covert toxocariasis [2].

The tests are available for the immunodiagnosis including enzyme-linked immunosorbent assay (ELISA) and Western blotting, both using *Toxocara canis *excretory-secretory (TES) antigens [3]. ELISA using TES antigens was the first method to be employed, irrespective of the clinical form of toxocariasis [4–6]. However, Magnaval et al. (1991) reported the high sensitivity and specificity of WB for immunodiagnosis of toxocariasis [7]. In addition, previous reports using both techniques indicated that Western blotting is a better method than ELISA to investigate patients suffering from visceral larva migrans or ocular larva migrans [8].

The epidemiological studies have noted the presence of *Toxocara* spp. infection in stray cats and dogs in various parts of Iran [9–11]. In Khorramabad, Iran, 22.2% of the public parks studied showed *Toxocara* eggs [12]. 

*Toxocara* is one of the common helminthic parasites reported that affect the human central nervous system (CNS). Several epidemiological and clinical studies have reported a correlation between *Toxocara canis* infection and epilepsy. Recently some authors have investigated this association in different geographic locations through case-control studies using serological tests [13–18].

To the best of our knowledge, there is no precise report from the anti-*Toxocara* antibodies in epileptic patients in the region. Therefore, this study afforded an opportunity to investigate the antibody response to *Toxocara* infection in epileptic patients by the performance of TES-ELISA and Western blotting.

## 2. Materials and Methods

### 2.1. Study Population

This study was conducted on 85 consecutive patients with idiopathic epilepsy, who were evaluated regarding their medical history, clinical characteristic, cranial imaging such as computed tomography (CT) scans, or magnetic resonance imaging (MRI) at the Neurology Division of Shohadaye-Ashayer Hospital from March to December 2011. Controls subjects were 85 volunteers from health care workers and the relatives of patients without history of neurological disorders. Epidemiological data determining the socioeconomic status of the participants groups were collected by a paper-and-pencil questionnaire. Neurological examination for each individual was performed, and the physical findings were recorded. 

### 2.2. Serological Tests

Serum samples of all participations were collected and stored at −20°C until used. Anti-*Toxocara* antibodies (IgG) were detected by a commercial Enzyme-Linked Immunosorbent Assay (ELISA) kit (IBL, International Gmbh, Hamburg, Germany) following the manufacture's guidance. Briefly, diluted sera samples (1 : 100) were added to wells; after 30 min incubation at 37°C, horseradish peroxidase-conjugated goat anti-human IgG was added at a 1 : 1000 dilution (30 min at 37°C), followed by the tetramethylbenzidine (TMB) substrate. Absorbance readings were made at 450 nm; a cut-off absorbance value was defined as the mean absorbance reading for three negative control sera plus two standard deviations. Antibody levels were expressed as reactivity indices, which were calculated as the cut-off value; positive samples had reactivity indices more than 0.553.

Immunodiagnosis by the Western blotting technique relied also upon a commercial kit (LDBIO Diagnostic, Lyon, France). In brief, the strips incubated for 2 h at room temperature, with sera at a dilution of 1/100. Before being washed at least three times in PBS containing 0.1% Tween 20, the strips were further incubated for 2 h at room temperature with the second antibody, anti-human immunoglobulin G peroxidase conjugate (dilution 1/1,000). After washing as mentioned previously, the substrate diaminobenzidine was added and the reaction stopped with several washes in distilled water. The results were considered as positive, when the samples react to two or more low-molecular-weight bands (LMWB; 24–35 kDa).

### 2.3. Statistical Analysis

We conducted statistical analysis using SPSS version 15.0 of windows 2003. Chi-squire test and Fisher's exact test were used for categorical data. A *P* value that is less than 0.05 was considered statistically significant.

### 2.4. Ethical Considerations

The study was approved by ethical committee of the Lorestan University of Medical Sciences, and informed consent was obtained from the participants prior to data collection.

## 3. Results

### 3.1. Characteristics of the Collected Pairs

Patients and control groups comprised 85 cryptogenic epileptic patients and 85 healthy persons, respectively. Of the patient group, 55 participants were males and 30 were females. The participants' ages ranged from 8 to 62 years old. Of the control group, 57 participants were males and 28 were females. These participants' ages in control group ranged from 7 to 59 years old. The epidemiologic and demographical factors in patients and healthy groups are shown in Table 1.

Neuroradiologic findings (CT scan or cranial MRI examination) were normal in 78.8% (*n* = 67) of the patients, whilst 21.8% (*n* = 18) had abnormal electrical activity in all of the cerebral cortices simultaneously. On the basis of the 1981 ILAE classification, 70 patients (82.4%) had partial seizure, out of whom 53 had secondary generalization and 17 were without secondary generalization.

### 3.2. Enzyme-Linked Immunosorbent Assay Testing

**Figure 1:**
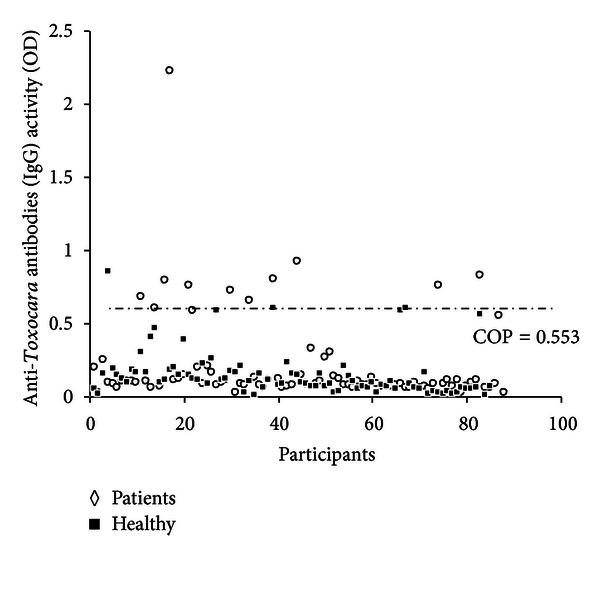
Dispersion of the anti-*Toxocara* IgG in serum samples of epileptic patients and healthy persons. Numbers in *X*-axis represent each sample (COP: cut-off point).

The population seroprevalence of *Toxocara* antibodies was significantly higher in the epileptic patients compared with the healthy group (Figure 1). Based on the ELISA results, 10 (11.8%) persons with epilepsy presented antibodies against *Toxocara* (Table 2). Among the seropositive patients, six (60%) were males and four (40%) females. These seropositive patient's ages ranged from 14 to 29 years old (mean: 19.8 ± 11.42). Of 85 healthy individuals, 3 (3.5%) were positive for *Toxocara* by initial ELISA screening. Among the seropositive persons in control group, 2 (66.7%) were males and one (33.3%) was female. According to the questionnaire response, patients were categorized according to their age groups and also living environment. The difference between presence of *Toxocara* infection and age groups of the patients was not found statistically significant (*P* > 0.05). The characteristics and distributions of the epileptic patients are shown in Table 3.

### 3.3. Western Blotting

**Figure 2:**
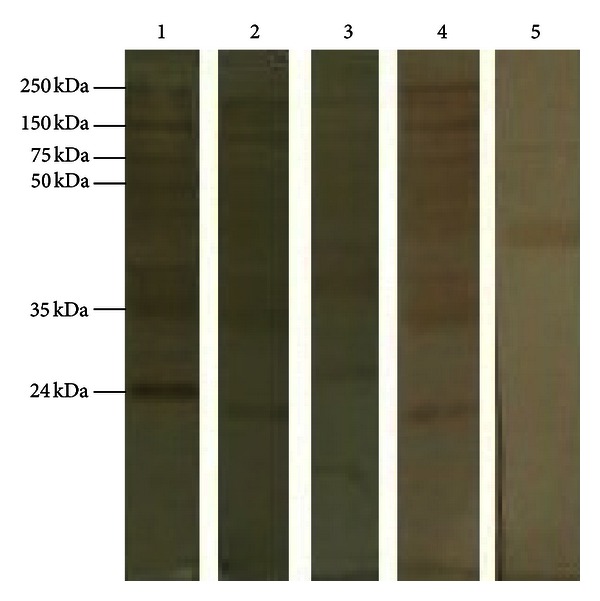
Western blotting results for sera sample of the study population. Lane 1: reference ladder; lanes 2–4: sera testing positive by ELISA; Lane 5: *Toxocara*-negative control.

Serum samples from patients and control groups that were positive for anti-*Toxocara* antibodies by ELISA were investigated using confirmatory Western blotting analysis. The positive Western blotting strips indicating the presence of specific anti-*Toxocara* IgG in the samples showed at least two or more bands of lower molecular weight between 24 and 35 kDa (Figure 2). Anti-*Toxocara *antibodies were detected in 3 (3%) ELISA positive patients, whereas all ELISA positive persons in control group were negative by Western blotting. Of 18 patients with abnormal neuroradiologic findings, 2 persons were seropositive by ELISA and immunoblot.

## 4. Discussion

Epilepsy is considered an important health problem in the developing countries [19]. A possible association between toxocariasis and epilepsy has been hypothesized, and *Toxocara* infection has been suggested as cofactor for epilepsy [13].

Serological studies conducted in various parts of the world demonstrate a variation in *Toxocara* seroprevalence ranging from 1.8 to 58.3 percent [20]. There are only a few studies reporting toxocariasis prevalence in Iran. A previous study has shown that the seroprevalence was 25.6% in school children [21]. We have previously reported that *Toxocara* eggs prevalence was 22.2% in public parks in Khorramabad, an urban region in Iran [12].

According to the literature research, neurological disorders in humans due to the presence of *Toxocara canis* larvae in the central nervous system might not be an uncommon event [22]. Despite the higher seroprevalence of *Toxocara canis* antibodies found in people with epilepsy versus controls in previous studies, there has been doubt about whether this implicated causality [13].

In this study, frequency of *Toxocara* infection in epileptic patients was 11.8%, scientifically higher than the healthy group (3.5%). Therefore, due to the reported prevalence of toxocariasis in the general population in Iran, the relatively low frequency of *Toxocara *infection in our control group required cautions interpretation. Of all the epilepsy patients seropositive for *Toxocara*, seven cases (70%) were males. However, this was not higher than the overall proportion of the male epilepsy patients of 64.7 percent. Toxocariasis is seen more frequently among children or young adults probably due to more frequent contact with infective eggs or by the ingestion of encapsulated larvae contained in the raw tissues of paratenic hosts, such as cows, sheep, or chicken. In the present study, we did not observe any significant difference between either of the seropositive and seronegative epileptic patients in terms of rural and urban populations and also various age groups. The seropositivity was not affected by age, although there were significantly more seropositive epilepsy patients who were students.

The diagnosis of human toxocariasis depends on serological test (ELISA) by using excretory-secretory antigens from *Toxocara* larvae, because it is very difficult to detect infective *Toxocara* larva in biopsy specimens. The antigens used in TES-ELISA are a complex mixture of glycoproteins, and cross-reactivity has been demonstrated with other helminthic infections (ascariasis, enterobiasis, trichuriasis, anisakiasis, taeniasis, echinococcosis, fascioliasis, and schistosomiasis). Therefore, ELISA and Western blotting are the most commonly used methods to determine anti-*Toxocara* antibodies [23–26]. Western blotting is the most sensitive and specific of the two assays available for immunodiagnosis of human toxocariasis. The high specificity of the test is based on the distinction between clusters of higher and lower molecular weight fractions. A previous study has showed that higher molecular weight bands are not specific and are suggestive of cross-reactions with other helminths, while lower molecular weight bands demonstrated a high level of specificity [7]. In this study, the immunoblot method that was applied to diagnosis of toxocariasis was more sensitive and specific than detecting by TES-ELISA. Therefore, our results confirm previous findings that the ELISA can be used for screening of patients depending on the specific antigen used and the *Toxocara* species [27].

## 5. Conclusion

Toxocariasis is a prevalent and treatable disease; the findings confirm that it could play an important role in the incidence of epilepsy in endemic areas of developing countries, and *Toxocara* infection could increase the risk of epilepsy that results from central nervous system disorders. On the other hand, results of our study demonstrate that a Western blotting based on TES performed a high specificity and is an appropriate confirmatory test for *Toxocara *monitoring in epilepsy patients. Serological monitoring in patients should follow a two-step testing scheme, that is, screening for *Toxocara* antibodies by ELISA followed by confirmation of seropositive reactions by means of Western blotting. 

## Figures and Tables

**Table 1:** The epidemiological and demographical factors in patients and healthy participants.

Factors	Patients, *n* (%)	Healthy, *n* (%)	Total	*P* value
Age group (years)				
<20	42 (49.5)	38 (44.7)	80	>0.05
20–39	28 (32.9)	34 (40.0)	62	
40–59	12 (14.1)	13 (15.3)	25	
≥60	3 (3.5)	0 (0.0)	3	
Sex				
Female	30 (5.3)	28 (32.9)	58	>0.05
Male	55 (64.7)	57 (67.1)	112	
Residence				
Rural	67 (72.8)	70 (82.4)	137	>0.05
urban	18 (21.2)	15 (17.6)	33	
Metier				
Student	37 (45.1)	22 (26.2)	59	<0.05
Worker	26 (31.7)	38 (45.2)	64	
Other	22 (23.2)	25 (28.6)	47	
Schooling level				
No school	13 (15.3)	21 (24.7)	34	>0.05
Some high	15 (17.7)	6 (7.1)	21	
High school	40 (47.1)	32 (37.6)	72	
Some college graduate school	27 (31.8)	26 (30.6)	53	

Total	85 (100)	85 (100)		

**Table 2:** Seroprevalence of antibodies to *Toxocara canis* in the epilepsy patients and healthy persons by ELISA.

	Epilepsy patients (%)	Healthy (%)	Total (*n*)
Seropositive	10 (11.8)	3 (3.5)	13
Seronegative	75 (88.2)	82 (96.5)	157

**Table 3:** Seroprevalence of *Toxocara *seropositive and seronegative in the epilepsy cases considering residency and age group.

	Seropositive, *n* (%)	Seronegative, *n* (%)	Total	Statistic
Residence				
Rural	7 (70.0)	60 (80.0)	67	Not significant
Urban	3 (30.0)	15 (20.0)	18	
Age group (years)				
<20	6 (60.0)	36 (48.0)	42	Not significant
20–39	3 (30.0)	25 (33.3)	28	
40–59	1 (10.1)	11 (14.7)	12	
≥60	0 (0.0)	3 (4.0)	3	
